# How Can We Help People With Long‐Term Health Conditions Understand and Engage in Physical Activity Maintenance From Healthcare to Community Settings? Perspectives of Patients, Carers and Professionals

**DOI:** 10.1111/hex.70961

**Published:** 2026-11-05

**Authors:** James P. Gavin, Luisa Holt, Xylo Chiu, Charlotte Dando, Suzanne McDonough

**Affiliations:** ^1^ School of Health Sciences, University of Southampton Southampton UK; ^2^ RCSI University of Medicine and Health Sciences Dublin Ireland

**Keywords:** community‐based, exercise referral, health inequalities, long‐term conditions, physical activity maintenance, primary care, self‐management

## Abstract

**Introduction:**

Physical activity (PA) is vital for managing long‐term health conditions (LTCs), yet maintaining regular PA remains a global challenge. Partnerships between healthcare, leisure, and community services can provide accessible PA opportunities, but current pathways focus on initiating, rather than maintaining PA. Understanding factors influencing progression along PA pathways and engagement of professionals is essential.

**Methods:**

Four focus groups of people with LTCs (n = 21; aged 39–82 years) were complemented by interviews with 19 professionals (aged 26–66) and five carers (aged 20–78 years). Inductive thematic analysis was used to generate understanding about recommendations for an integrated PA maintenance pathway, before mapping themes to the COM‐B (Capability, Opportunity, Motivation, Behaviour) model.

**Results:**

Key themes were: i) limited access to, and information about PA services; ii) a need for improved intersectoral collaboration; and iii) opportunities and barriers to developing a PA maintenance pathway. Primary care and community centres were deemed reliable and accessible locations to advertise local opportunities, but without digital platforms, lacked recency and condition‐appropriate activities. Intermediaries emerged as community navigators, working intersectorally with knowledge of local communities, and behaviour change techniques to support maintenance. However, funding cuts limited intersectoral working.

**Conclusion:**

Maintaining PA among people with LTCs and carers requires a coordinated, intersectoral pathway between healthcare and community services, which extends beyond initial consultation and referral. Investment in collaborative infrastructure, accessible digital systems, and sustainable funding is lacking. However, community‐based intermediaries trained in digital tools, can play a pivotal role in supporting maintenance by connecting individuals with appropriate opportunities and providing ongoing behaviour change.

## 1 Public and Professional Contribution

Public involvement began in the planning stage, via a series of knowledge exchange community cafes, which aimed to engage people living with LTCs, and their unpaid carers at a city‐level, including those from deprived and underserved communities (Supporting material 1). Cafes helped also helped in study recruitment. Professional involvement occurred alongside this work via the research team's networks in academia, health and social care, and local authorities. Professional involvement helped identify additional healthcare and community services, involved in promoting PA for LTC communities. Individuals were not reimbursed for their community cafes, as these were public engagement activities. Public involvement in this project comprised: engaging underserved communities in PA education across an urban locality; facilitating involvement in research design (e.g., preference for in‐person focus groups, rather than online); and results interpretation (e.g., sharing themes with participants for feedback).

## 2 Introduction

The World Health Organisation (WHO) estimates that around two‐thirds of deaths globally (~38 million) are due to long‐term health conditions such as cardiovascular disease, diabetes, cancer, and chronic respiratory disease [1]. Long‐term conditions (LTCs) can be prevented and managed by modifiable individual lifestyle and behavioural factors, such as physical activity (PA), healthy diet, and alcohol and tobacco intake [1, 2]. Physical activity has particular importance in LTC management, as it benefits physical [3], mental [4] and social health [3], as well as reducing healthcare costs [2]. However, ~30% of the global population (1.8 billion) are physically inactive according to WHO guidelines [5], and for those living with LTCs, the prevalence is even greater [6, 7], particularly for those residing in socioeconomically deprived areas [7, 8].

Intersectoral collaboration between health and social care, leisure, local government and community services are becoming cost‐effective, whole system approaches to support population‐level PA in the prevention and management of LTCs [9, 10]. Healthcare professionals are pivotal first contacts within these to help patients manage their own conditions, and with brief interventions, support people in becoming active in their communities [6, 11, 12, 13]. Within many national health systems, physical activity referral schemes (PARS) advance upon this, with the healthcare professional assessing the patient, before referring to exercise specialists [14, 15].

The effectiveness of PARS globally is notoriously difficult to determine, due to the complexity of schemes, differing components [16, 17], and heterogeneity of evaluation data [15, 18]. In Australia for example, exercise professionals operate across public and private healthcare, supporting people with LTCs across community‐based primary care, occupational rehabilitation, and privately funded settings [19]. Whereas UK PARS are established in publicly funded primary care settings, with referral to local council funded and privately funded community‐based PA [20], and in Germany, not yet implemented nationwide [21]. Accredited exercise professionals possess the knowledge and skills of personalised, exercise prescription [22], yet lack training in behaviour change, and in clinical settings their implementation is unstandardised and inconsistent [23, 24]. As many nations move towards neighbourhood health, whereby pathways between healthcare and community services are strengthened to provide care at a local level [25, 26, 27], ‘intermediaries’ such as social prescribers, health promotion officers and health coaches are emerging as capable of supporting people with LTCs in positive lifestyle changes [28], such as maintaining PA into community living [29, 30].

Little understanding exists of how to operationalise and implement whole system, intersectoral collaboration to promote long‐term maintenance of PA [6, 9, 10]. Emerging evidence suggests that co‐production and engagement with public and professionals is crucial when developing, implementing and evaluating intersectoral pathways to promote PA [13, 19, 21, 31]. These, however, primarily focus on increasing PA, rather than maintaining PA over the long‐term for LTC prevention and management. For example, the UK National Institute of Healthcare Research (NIHR) ‘Moving Matters’ [32] emphasises the importance of providing appropriate opportunities for PA, to develop people's motivations and capabilities in becoming active. Based on the COM‐B model [33], which suggests that ‘Capability’, ‘Opportunity’ and ‘Motivation’ influence an individual's ‘Behaviour’, insights from PA programmes for people with LTCs demonstrate that peer and professional relationships both motivate, via encouragement and accountability, and provide opportunities to increase PA [14, 31, 34], but fail to develop autonomy and habit formation for long‐term behaviour change post‐intervention [14].

We previously identified five operational components necessary for a successful pathway to maintain PA behaviour change: i) recruitment and referral; ii) triage and personalisation; iii) behavioural support; iv) appropriate PA opportunities; and v) signposting to ‘exit routes’ and/or ongoing support [14, 35]. At a regional level, we also revealed complex inequalities in participation (with PARS participants predominantly white, educated, retired and physically active), and a need for improved intersectoral partnership working [14]. The optimum processes and strategies for connecting people with LTCs across the socioeconomic spectrum [36], and with more complex health needs within an intersectoral pathway, remain unclear [29].

To help reduce inequalities in PA for people with LTCs, the UK Office for Health Improvement and Disparities [36] have developed evidence‐based resources to equip health and social care professionals, in specific activities and interventions to reduce physical inactivity; engage and collaborate with communities; and identify community‐based resources and services to promote PA for people with LTCs. Aiming to promote PA and engage underserved communities, we advanced our preceding research, which identified the support needs of people living with LTCs, exiting exercise referral schemes across the region [14]. We sought to advance our earlier work by gaining insights for LTC communities at a local, city‐wide level, including areas with substantial health inequalities. This research aimed to identify and understand the factors influencing:

i. Progression along an intersectoral pathway between healthcare and community services to support PA maintenance for people with LTCs and carers, and;
ii. Multisectoral professionals involved in developing, delivering or supporting access to these PA services.

Specific focus was on the enablers and barriers for maintenance of PA for LTC participants from an intersectoral perspective between healthcare, leisure and community services. Hereafter, intersectoral refers to between sectors; multisectoral refers to involving or relating to multiple sectors.

## 3 Methods

### 3.1 Research Design

This study employed a qualitative exploratory design using inductive thematic analysis to understand the experiences of individuals with LTCs and their carers, alongside the perspectives of multisectoral professionals in the culturally diverse city of Southampton, England, UK. Collaboration with local government, healthcare and community organisations directly informed the aims, design, and recruitment of this research, including interview guide development and gatekeeping for professional interviews. These organisations promoted PA and policy development across the city and/or region, and included Move Consulting, a national PA management organisation, co‐leading the development of the UK guidance for creating PA pathways. Ongoing consultation with them ensured that the research aligned to regional and national agenda for population‐level PA promotion.

To recruit people living with LTCs, carers and multisectoral professionals across diverse communities at a local, urban level, we collaborated with Southampton City Council, and Energise Me [37], the Hampshire and Isle of Wight Active Partnerships Organisation, to deliver a series of community cafés. Active Partnerships are non‐profit organisations funded by Sport England, that seek to collaborate with local authorities, healthcare sectors, and charities to promote PA, helping to reduce health inequalities [38]. Four cafés were held at a Baptist church (located in the top 10% of most deprived neighbourhoods in the country), three at a football stadium (top 10‐20% deprived), two at a housing association (top 20% deprived), one at a leisure centre (top 30% deprived), and one at a homecare residence (top 40% deprived) [39] (Mar‐Aug 2024; Figure 1).

**Figure 1:**
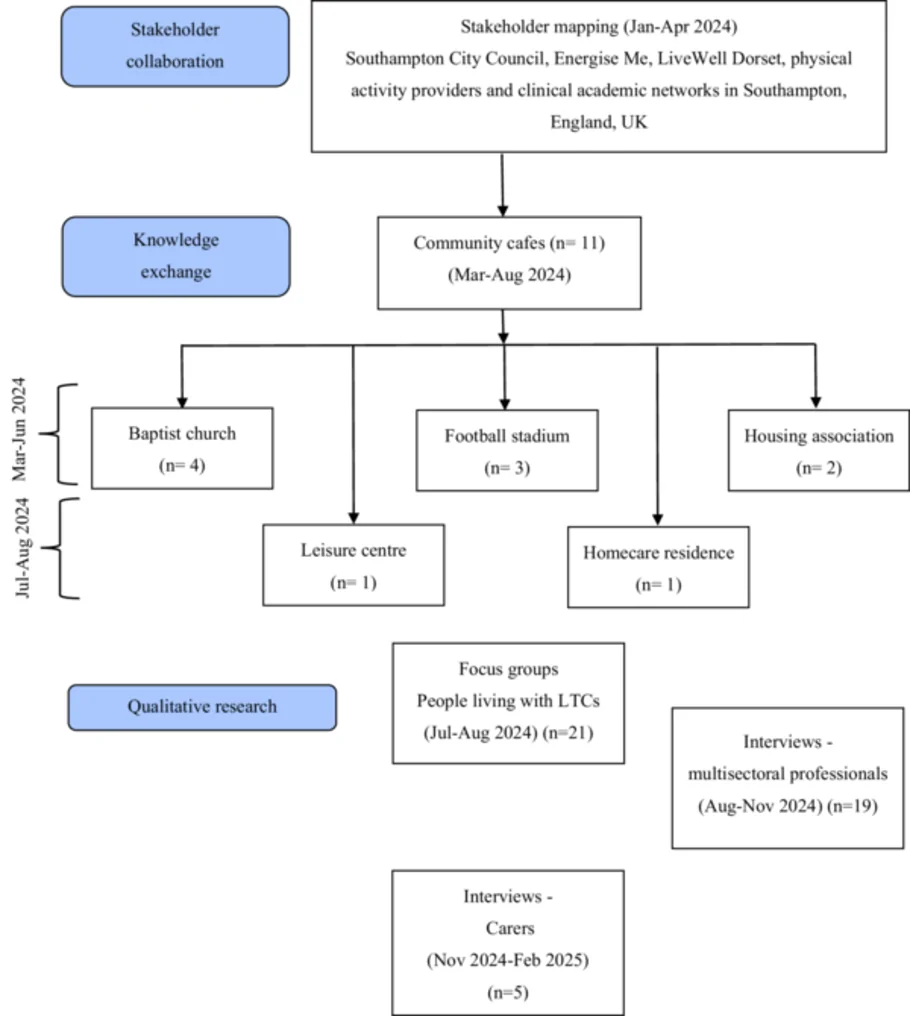
Project flow diagram – phases and recruitment. Adapted from CONSORT 2025 reporting guidelines [40].

Cafes allowed us to share recent work on PA maintenance from a regional, PARS‐specific level [14, 41] recruit at a local, community level, allowing a targeted approach to understanding diverse support needs for people with LTCs, carers and professionals beyond PARS, and across wider community schemes. Data were not collected in community cafes, as these were knowledge exchange activities to engage, involve and recruit public living with, and caring for LTCs to the qualitative research.

Following the community cafés, qualitative focus groups of people with LTCs, and semi‐structured interviews with carers and multisectoral professionals were conducted, framed using the COM‐B model [33] ([Supporting material 2]‐4), and reported to the COREQ guidelines [42] ([Supporting material 5]).

### 3.2 Sampling and Recruitment

Convenience sampling, coupled with maximum variation sampling, allowed for recruitment of people with LTCs and carers from the community cafés across the socioeconomic strata (Apr‐Jul 2024). Recruitment media were advertised at cafés and across the networks of multisectoral partners. Purposive sampling was initially used to recruit professionals across healthcare, leisure and community sectors, involved in developing, delivering and/or supporting access to PA services for people with LTCs (May‐Jul 2024). Thereafter, snowball sampling from professional's contacts allowed for balanced representation in key characteristics (e.g., profession, sector, and LTC specialism) (Jun‐Oct 2024).

Potential participants completed a brief screening form to determine eligibility, which included key demographic characteristics (e.g., role, sector [professionals]; age, ethnicity, habitual PA [people with LTCs and carers]). Eligibility for participants with LTCs were: aged ≥ 18 years, diagnosis of ≥ 1 LTC, and residence of Southampton city or bordering locality. Carer eligibility included: unpaid caring responsibilities for a partner, family and/or friend living with one or more LTCs. Professional eligibility included: having ≥ 3 years’ experience of working in organisation with responsibility for developing, delivering and/or supporting PA for people with LTCs in healthcare, leisure or community sectors. Ethical approval was granted by the University of Southampton's Faculty of Environmental and Life Sciences research ethics committee (ERGO ref: 92231). Consent was obtained from participants prior to interviews and focus groups, which were audio‐recorded and transcribed verbatim, with participants anonymised on transcription.

### 3.3 Data Collection

Focus groups involving people with LTCs lasted 50‐70 min, were held in‐person or online, from July and August 2024. Carer interviews lasted 30‐50 min in‐person, from November 2024 to February 2025. Focus groups and interviews followed semi‐structured guides, focussing on the participant's experiences of maintaining PA, broadly relating to capability (psychological and physical), opportunity (social and physical) and motivation (reflective and automatic), whilst living with, or caring for someone with a LTC(s). Interview guides were informed by our earlier work, highlighting a need for improved intersectoral partnership [14], developed based on the COM‐B model [33] (barriers and enablers), and Move Consulting's key PA pathway components (Supporting material 2) [35]. In‐person focus groups and interviews were held in community settings across the city, audio‐recorded and transcribed verbatim. Online groups and interviews were held, audio‐recorded and automatically transcribed via Microsoft Teams. JG facilitated each public focus group/interview, with XC taking fieldnotes to describe group dynamics, social context and reflections on completion.

Online interviews were conducted with professionals using Microsoft Teams from August to November 2024. Interviews lasted 45‐60 min following a guide designed to discuss the professional's experiences of supporting PA behaviours for people with LTCs, which included intersectoral collaboration. In adopting the COM‐B model [33] for multisectoral professionals, we discussed the physical and psychological capabilities, social and physical opportunities, and motivation of their LTC clients in maintaining PA (Supporting material 3). For all groups, their experiences were discussed in‐light of enablers and barriers for PA maintenance. The initial six professional interviews were conducted by the lead researcher, LH, of the preceding study [14], who briefed two researchers to conduct the final 13 interviews (JG and XC). Microsoft Teams transcripts were verified for all cohorts within 36 h of the interview or focus group, with one researcher (XC) checking each automated transcript against the live recording.

People with LTCs, carers and professionals were also asked about their preferences for an intersectoral pathway between healthcare, leisure and community services, to help people with, and carers for LTCs maintain PA, long‐term (Supporting material 4). This knowledge, initially created ‘bottom‐up’ inductively, was subsequently aligned to the COM‐B, to help early co‐design for future intervention. Data saturation was defined as no new information or data richness, emerging from each professional sub‐sample (e.g., health, social care, local government) or focus group [31].

### 3.4 Data Analysis

Thematic analysis was used inductively [43], as transcripts were coded line‐by‐line, and then grouped to sub‐themes and themes. Themes were then deductively mapped to the COM‐B model, specific to enablers and barriers [33] for PA maintenance. Interviews and focus groups were transcribed by XC and imported to NVivo (release 14.23.4) for analysis. Three researchers (LH, JG, XC) independently coded the first two transcripts, before meeting to compare and refine coding [31], with an independent researcher (CD) resolving disagreements. Thereafter, LH coded a further four transcripts for the professional interviews, whilst JG and XC coded the remaining 23 transcripts, with guidance from LH to shape the coding map and check agreement.

Coding involved transcripts being read and re‐read line‐by‐line, independently by the researchers in NVivo, before initially generating and grouping codes in NVivo. The two researchers checked their coding together, with researcher, LH, resolving disagreements. Initial reflections were noted individually, with collaborative discussions ongoing involving the independent researcher (LH), to share meaning and gain consensus on coding. Next, the lead researcher (JG) mapped the higher‐level themes and related subthemes offline, using the ‘One Sheet Of Paper’ approach [44] onto A3 paper. Paper mapping was conducted for each cohort, as a method of synthesising and visualising themes that were emerging from the software‐based coding. Once each thematic map was drafted, it was sent to the research team for feedback and refinement, which included focusing on differences and similarities between people with LTCs, carers and professionals.

Finally, the three thematic maps were combined by two researchers (JG, XC), who refined themes by reducing overlap of concepts and ensure a full narrative from the data. The same researcher (LH) was again consulted to gain consensus in subtheme and theme refinement, which resulted in three core themes. Subthemes from these themes were then deductively mapped to the COM‐B model, according to whether they related to factors affecting capability, opportunity, motivation of the behaviour. In beginning to co‐design an intersectoral pathway to support PA for people with LTCs, this approach provided insight into factors influencing behaviours and behaviour change, for LTC participants, their carers, and the professionals supporting them.

## 4 Results

### 4.1 Participant Demographics

Four focus groups of people living with LTCs (n = 21; aged 43‐81 years), five interviews with carers (aged 20‐78 years; Table 1), and 19 interviews with multisectoral professionals (aged 26‐66 years; Table 2) were conducted. People with LTCs were primarily white British (67%), female (76%), with eleven retired, at least eight living with multimorbidity, and most self‐reporting as physically inactive (57%). Carers shared similar demographics but considered themselves as moderately active (80%). For participants living with LTCs and their carers, 35% lived in the most deprived 10% of neighbourhoods, whereas 62% lived within the neighbourhoods with moderate‐to‐high levels of deprivation (Table 1).

**Table 1:** Demographics of participants living with long‐term conditions (LTCs) and carers.

Demographic		Total sample (n = 26)	People with LTCs (n = 21)	Carers (n = 5)
Age (years; mean ± SD (range))		59.9 ± 13.4 (20‐81)	61.3 ± 11.3 (43‐81)	54 ± 21.6 (20‐78)
Sex (n (%))	Male	7 (27%)	5 (24%)	2 (40%)
	Female	19 (73%)	16 (76%)	3 (60%)
Ethnicity (n (%))	White British	18 (68%)	14 (67%)	4 (80%)
	Asian	2 (8%)	1 (5%)	1 (20%)
	Black Caribbean	2 (8%)	2 (10%)	0 (0%)
	White (Irish)	2 (8%)	2 (10%)	0 (0%)
	Other (Indian Asian)	2 (8%)	2 (10%)	0 (0%)
Domestic status (n (%))	Married/living with a partner	11 (42%)	8 (38%)	3 (60%)
	Single	5 (19%)	5 (24%)	0 (0%)
	Divorced/separated	4 (15%)	3 (14%)	1 (20%)
	Widowed	6 (23%)	5 (24%)	1 (20%)
Highest level of education (n (%))	University degree	9 (35%)	6 (29%)	3 (60%)
	Further secondary (college)	8 (31%)	7 (33%)	1 (20%)
	Secondary	5 (19%)	5 (24%)	0 (0%)
	Other professional qualification	4 (15%)	3 (14%)	1 (20%)
Neighbourhood level of deprivation* (n (%))	Top 10% most deprived area	9 (35%)	8 (38%)	1 (20%)
	2nd – 4th centile	7 (27%)	6 (29%)	1 (20%)
	5th – 6th centile	3 (12%)	1 (5%)	2 (40%)
	7th – 9th centile	5 (19%)	5 (24%)	0 (0%)
	Top 10% least deprived area	2 (8%)	1 (5%)	1 (20%)
Employment status (n (%))	Full‐time	1 (4%)	1 (5%)	0 (0%)
	Part‐time	1 (4%)	1 (5%)	0 (0%)
	Retired	15 (58%)	11 (52%)	4 (80%)
	Unemployed	1 (4%)	1 (5%)	0 (0%)
	Disabled (part‐time)	2 (8%)	2 (10%)	0 (0%)
	Disabled (unemployed)	3 (12%)	3 (14%)	0 (0%)
	Other (volunteer, semi‐retired)	3 (12%)	2 (10%)	1 (20%)
Primary LTC** (n (%))	Musculoskeletal	10 (38%)	9 (48%)	1 (20%)
	Type ii diabetes	8 (31%)	6 (29%)	2 (40%)
	Cardiovascular	5 (19%)	3 (14%)	2 (40%)
	Neurological (i.e., stroke, CFS)	2 (8%)	2 (10%)	0 (0%)
	Respiratory (COPD)	1 (4%)	1 (5%)	0 (0%)
Confirmed multimorbidity		10 (38%)	8 (38%)	2 (40%)
Current level of physical activity (PA) participation†	Active	5 (19%)	4 (19%)	1 (20%)
	Moderately active	9 (35%)	5 (24%)	4 (80%)
	Insufficiently active/sedentary	12 (46%)	12 (57%)	0 (0%)

*Level of deprivation for an individual's local area, based on the 2025 English Indices of Deprivation [39], from 1st (top 10% most deprived) to the 10th centile (top 10% least deprived). **Primary LTC for which they were referred to an activity scheme. All (n = 26) from Hampshire, UK; †Self‐rated using the Godin Leisure‐Time Questionnaire [45]. CFS, chronic fatigue syndrome; COPD, chronic obstructive pulmonary disease; LTC, long‐term condition; PA, physical activity.

**Table 2:** Demographics of multisectoral professionals.

#	Profession	Sex	Sector	Role description ‐ service context	Experience supporting LTCs (yrs)
1	Health and wellbeing coach	Female	Health and social care	Healthcare ‐ primary care and community (intermediary)	4
2	Health and wellbeing coach (team lead)	Male	Health and social care	Healthcare ‐ primary care and community (intermediary)	12
3	Manager ‐ local charity	Female	Voluntary, community	Social care ‐ promote social connections for people at risk of loneliness and isolation	16
4	Community development worker	Male	Voluntary, community	Community services ‐ facilitate activities and services (intermediary)	10
5	Parkrun director	Female	Voluntary, community	Voluntary services ‐ provision of community‐based physical activities	13
6	Health and personal trainer	Male	Leisure	Healthcare ‐ clinical exercise trainer	8
7	Carer support worker/dementia ambassador	Female	Health and social care	Social care ‐ support for unpaid carers	28
8	Manager ‐ mental health charity	Female	Voluntary, community	Community services ‐ provision of community occupational activities	15
9	Public health consultant	Female	Local government	Healthcare ‐ policy setting and implementation	12
10	Sports facilitator	Male	Leisure	Leisure services ‐ provision of community‐based physical activities	27
11	Contract partnerships manager	Female	Leisure	Leisure services ‐ provision of physical activity referral schemes (PARS)	18
12	Manager – healthcare charity	Female	Voluntary, community	Community services ‐ policy setting and implementation	18
13	Personalised‐care team lead	Female	Health and social care	Healthcare ‐ care planning within UK NHS primary and community care services	4
14	Social prescriber	Female	Health and social care	Social care ‐ primary care and community (intermediary)	4
15	Parkrun director	Male	Voluntary, community	Voluntary sector ‐ provision of community‐based physical activities	12
16	Social prescribing service co‐ordinator	Male	Voluntary, community	Social care ‐ primary care and community (intermediary)	—
17	Physiotherapist	Male	Health and social care	Healthcare ‐ provision of clinics for people with LTCs	8
18	Community activist	Female	Voluntary, community	Voluntary services ‐ community support for diverse groups	5
19	Team lead/advanced clinical practitioner	Female	Health and social care	Healthcare ‐ care planning within UK NHS primary and community care services	25

Data missing from P#16. LTC, long‐term condition; NHS, National Health Service; UK, United Kingdom.

Professionals worked across health and social care (n = 7), voluntary, community (n = 8) and leisure sectors (n = 3), and local government (n = 1) (Table 2). Most (60%) had supported people with LTCs for ≥ 10 years, by recommending PA (21%), developing PA services (14%), recruiting or referring (5%), triage or personalisation (10%), and providing PA delivery (15%), providing signposting/ongoing support (18%). Only 17% regularly delivered behaviour change techniques, such as goal setting and coping planning to support PA maintenance. Support provision ranged from providing written information, advice and activities, to referrals and signposting, to providing transport and carer‐specific support.

Three core themes were identified, relating to progression along an intersectoral pathway between healthcare and community services: i) limited access to and information about PA services; ii) a need for improved intersectoral collaboration; and iii) opportunities and barriers to developing a pathway for PA maintenance. Participants are descriptively labelled in quotes according to their cohort, where LTC = person with LTC(s), P = professional, and C = carer.

## 5 Theme 1. Limited Access to and Information About Appropriate Physical Activity (PA) Services to Support Transition

### 5.1 Experiences of People With LTCs and Carers

People with LTCs spoke about fluctuating physical capability due to condition symptoms, which influenced their motivation to remain active. For example, pain and fatigue symptoms, and managing multimorbidities were the main physical barriers impacting their engagement in PA. An individual's symptoms restricted their opportunities to engage in structured activities, which tended to demotivate and overwhelm participants.

…doing normal day‐to‐day tasks I find really tricky and challenging…when I'm also dealing with increased pain levels trying to engage in some kind of arranged activity is just really overwhelming. I end up retreating and not doing anything… FG1

(LTC, 62 years, female, type II diabetes/osteoarthritis)

For this reason, some participants recognised the importance of being independent and autonomous in staying physically active. For example, one carer raised that PA motivated them, as it afforded time for reflection and personal feedback, but was also necessary as caring duties restricted engagement in group physical activities. Time in managing caring, work and self‐care duties were the main barriers for carers.

…part of my caring responsibilities… wake up, I go and see her, I get her up, get her dressed, get her washed, get her fed. If I have the energy to go for a swim and then I go back, do more feeds, do more self‐care and then put her to bed at the end of the day and sort out cleaning… C5

(20 years, female)

These greatly reduced group PA opportunities, particularly for carers. Consequently, some carers and people with LTCs reported using incidental physical activities, such as taking the stairs, cleaning, and walking errands to sustain their PA. Scheduling and goal setting strategies (e.g., ‘If‐Then’ plans) were valued by people with LTCs and carers, particularly in maintaining PA whilst managing fatigue, flare ups, or in times of greater care provision.

Trying to moderate and keep it small scale… don't sort of be too ambitious with yourself. Just do small scale steps and you're more likely to do it. So just go for a shorter walk or spend less of time in the gym. You know… it's more achievable than if you're setting yourself a massive goal. FG2

(LTC, 52 years, female, musculoskeletal condition)

However, most participants favoured group classes offering both professional and peer social support. For people with LTCs, attending public exercise classes that were not targeted at clinical groups, could be isolating and demotivating, due to mismatched physical capabilities. This included being physically active with friends not living with LTCs. Many experienced being ‘prescribed’ inappropriate or intolerable exercises, which demotivated and ‘disengaged’ them. Due to these challenges, people with LTCs required age‐ and condition‐appropriate activities, particularly group classes emphasising both movement and social, peer interaction. This included chair‐based exercise classes, which a number advocated due to the social and functional benefits, of performing safe, professional‐led activities with their peers.

… we ended up having to do some simple workouts … sitting up and down off the chair. I could feel the muscles, you know, just by doing simple painting [work] when at home. It's making it, you know functional. You can walk around a little [chat] … and that's why chair base is powerful because when you've got operations in hospital, can you get out of bed? A chair? Then can you walk up and down the corridor. C2

(58 years, female)

Balance, increased falls risk, and disturbed sleep due to medications, were common challenges for many, which directly limited an individual's opportunities. Lack of sleep, in particular, had negative impact on people's energy and motivation levels for those managing LTCs in full‐time employment. Tiredness and fixed work schedules restricted where and when people with LTCs could be physically active. More broadly, maintaining healthy weight was raised as an important health issue, relating to general physical and mental health and mental wellbeing. Due to these biopsychosocial factors, awareness of their own condition(s) and reflection on their physical capabilities were valued by people with LTCs and carers, alike.

Keeping physically active keeps your mind active … it makes you feel healthier and that you should do better. You know it makes you want to eat better, it makes you want to look after yourself. C5

(20 years, female)

### 5.2 Need for Ongoing Support

Limited signposting to PA services from healthcare and exercise professionals was deemed an issue, particularly at the end of PARS and other structured, supervised programmes. Participants with LTCs believed that primary care settings were most suitable for signposting to, or promoting local opportunities, but felt these were not readily accessible due to not being prioritised/high clinical workloads, and a lack of continued, ongoing support and follow‐up (see Supporting material 6).

And there was almost nothing from the NHS in the way of advice or support like that. They simply said ‘Come back to us if you have a problem. We'll get in touch in 6 months to see how you're doing’. Which they didn't. FG2

(LTC, 57 years, female, type ii diabetes)

People with LTCs and carers felt ongoing support was necessary, particularly to maintain PA beyond structured, supervised programmes, supported by either community‐based healthcare or exercise professionals. Ongoing support needs differed for people with LTCs and carers. People with LTCs required support targeted at enhancing their capabilities, whereas carers required who required support targeted at improving access to opportunities.

### 5.3 Approaches to Improve Community‐Based Opportunities for Physical Activity (PA)

People wanted options and signposting to existing, appropriate physical activities in their communities, that were within their physical capabilities and did not aggravate symptoms. A potential solution raised by professionals was having both clinical and self‐referral options, to improve access and support people's health and PA needs. To support transition between services, one health coach suggested having skilled and knowledgeable healthcare and exercise professionals in consultations, equipped with appropriate communication skills and understanding of health conditions.

From a clinician who's sat on the chair, they're face‐to‐face with the patient, I think their confidence and their skills to have conversations is really important. I had a referral from a GP recently…a real prime example of a GP saying ‘I don't know where to start and what to advise them’…I did find some resources, which were designed for GPs in consultation to, if your patient has RA [rheumatoid arthritis] or COPD, then this is how you would have that conversation. It was lovely because it even broke it down into if you had 2‐min, 1‐min, 5‐min. P2

(health and wellbeing coach)

Appropriate activities offered confidence and insights into the range of suitable, community‐based PA options available for people with LTCs and their carers. GP surgeries, pharmacies and public libraries were deemed the most effective places to advertise community‐based opportunities for PA within individual neighbourhoods, this included their online websites or hubs. However, ‘community hubs’ – integrating together health and social care, voluntary services and local authorities, were valued as central locations by people with LTCs and professionals, to bring people together in sharing opportunities, and experiences of living with or caring for LTCs. Professional ‘intermediaries’ felt that information provision was key to improving public awareness, and that their involvement in community hubs could maintain their professional local knowledge, and promote intersectoral networking to share resources and opportunities.

If we can provide them [patients] with as much information as possible, then we're able to answer any question that the patient comes to us with… we're able to help increase their confidence. I think it's about us as professionals and having that network to be able to go out and talk to these providers, to go to those sessions, to get all the answers to these questions, as much as it is for the providers to create this information. P1

(health and wellbeing coach)

However, healthcare and exercise professionals not involved regularly in community, neighbourhood hubs were less familiar with local opportunities for signposting.

### 5.4 Approaches to Improve Online Opportunities for Physical Activity (PA)

People with LTCs valued expert guidance when using digital tools to manage their health and conditions online, and felt professionals expressed similar in providing care. This included frustration with regular system updates, which necessitated relearning, and often required personal support not readily available for those lacking capability.

…when things change, e‐Consult to Admiral or whatever it's called now. Some people don't use technology that often. I mean [I am] mixed generation, but even I find it frustrating using that stupid system, not being able to ring the doctor to do something for you, rather than just say, ‘do you want me to talk you through it?’ Which must be frustrating for them. FG4

(LTC, 68 years, male, type ii diabetes/COPD)

From the professional perspective, they saw a common ‘digital divide’, for which, older adults with LTCs were more vulnerable. A potential solution to this raised by a healthcare professional (physiotherapist) was peer‐to‐peer learning, to share and maintain digital skills. Intermediaries like health coaches and social prescribers were deemed best‐placed to start this, by signposting and training individuals in digital tools in their neighbourhoods. Online hubs with interlinking digital tools, such as exercise prescription software, were another solution to enhance intersectoral and public communications proposed by professional participants.

…particularly exercise prescription software that allows the patient to communicate back, can be really helpful. P17

(health and social care)

## 6 Theme 2. A Need for Improved Intersectoral Collaboration Within a Pathway

### 6.1 Improved Intersectoral Communications

Professionals reported challenges in sectors communicating in a timely and consistent manner, this included community organisations not having established networks within the city and streamlined/efficient communication pathways (Supporting material 7). Health and social care professionals reported how they had created local and city‐wide networks involving intermediaries, to help share knowledge and expertise, to enhance personalised care to their LTC patients. One healthcare professional mentioned system and administrative checks delayed working with intermediaries in the community, even within specific localities.

I needed to get the sign off first from the GP partners… So that's where it can slow down a little bit, back and forth, just checking a few things over. Again, different surgeries do very different things, which is quite surprising because they're all in the same area. P13

(health and social care)

### 6.2 Importance of Established and Trusted Links With Intermediaries

Most people with LTCs and carers were unaware of intermediaries, such as health and wellbeing coaches. Equally, some leisure professionals were unaware of the scope of newly established intermediaries, such as social prescribers. For those that were aware, they were highly positive of their role, especially in increasing interprofessional knowledge.

I think having more knowledge of what is around the ciy and what things are going on. One of my managers invited me to go to a green social prescribing event a few weeks ago in the town centre, and I met [name] who works for [organisation], and he was talking about the things they're doing. I wasn't aware that they've got a physical activity social prescribing course you can do and networking with him meant that I'm going to do that course…it's about who you know, rather than what you know. P6

(health and personal trainer)

### 6.3 Community Partnerships – Understanding the Experiences of Services and LTC Communities

Embedding PA into community groups and initiatives was deemed important to raise awareness of opportunities and promote inclusive involvement. Within this, peer development schemes were seen as a feasible solution to help maintain motivation, through peer‐to‐peer monitoring and accountability to others, if supported professionally. Volunteering was an example of this, whereby some professionals suggested training service users as volunteers, to promote: peer‐to‐peer learning, trusted partnerships with LTC communities, and sustainability in service provision. Having check‐ins from the programme providers were also important for people with LTCs and carers to support PA maintenance.

People have long‐term conditions, but people have so much to offer … the great thing about volunteering is that you are immediately helping others and you're being positive and doing something really useful. That switch of mindset is outstanding, you can make a massive difference to people's lives. They suddenly get back a sense of who they are and what they're about…. P4

(voluntary, community)

### 6.4 Understanding Experiences of Other Services and LTC Communities

Intersectoral, interdisciplinary working was crucial to optimise holistic care, as professionals could share and contribute specific expertise (e.g., mental health).

We work purposefully with around about up to a hundred different organisations. So we may be looking after shared people and they need different areas of specialism. P3

(voluntary, community)

From a workforce perspective, professionals with good local knowledge of services, opportunities and local geography, were seen to be better equipped to understand the specific challenges, needs, and support strategies for maintaining PA, in their LTC communities. e.g., providing people with LTCs and their carers with opportunities for volunteering, which was seen to enhance people's capabilities and self‐identities, whilst support local voluntary and community organisations. From a public perspective, people with LTCs and carers wanted both physical and digital media, to share experiences and opportunities for professional support.

## 7 Theme 3. Opportunities and Barriers to Developing a Pathway for Physical Activity (PA) Maintenance

### Effect of Limited Funding and Resources on Opportunities

The workload pressures facing healthcare professionals meant that people living LTCs were often unable to access services in a timely manner, and instead sought to self‐fund private healthcare to prioritise PA. This was partly attributable to reduced public funding, which limited the collaborative working that multisectoral professionals could do. However, for some it stimulated innovations in sharing resources and partnership working, to exchange expertise and knowledge ([Supporting material 8]).

…community navigation…we have a system that works…essentially, it's vastly underfunded. So, there's not many of us left doing it as it were, and that's been a process of cutbacks with the Council, particularly, not having any money to fund these services. So, we don't have to reinvent the wheel as it were, it does happen already, and we are doing it and it works but we just need more of it. Essentially, we need more resource to be able to do that. P4

(voluntary, community)

Regardless, people living with LTCs and carers were willing to subsidise PA, if they involved guided supervision, and were flexible to the individual's daily lives and routines.

… don't mind paying for things, but sometimes you have to pay for a term … or you have to pay if you're going to a certain session. You can't just … drop in. You know, there are quite a lot of free things about and that is amazing don't get me wrong, I think that's really good and you don't expect everything to be free, but you don't want to then pay for a term of something. C1

(64 years, female)

### 7.1 Digital Literacy

It is cost‐effective for resources to be online, but burdensome for some older participants who recognised the necessity, but also the distraction and time taken to navigate online. People with LTCs saw the importance of healthcare professionals and intermediaries being digitally trained, to raise awareness of, support engagement in and increase access to community‐based PA.

If it was on an app, then obviously, social prescribers or GP would be like ‘have you checked this out?’ How do you check that out? And then obviously the onus is on you, but at least they've told you where to look. FG4

(LTC, 48 years, female, COPD/cardiovascular condition)

A viable and cost‐effective approach to involve digital tools in PA programmes, raised by a participant with LTCs, was to commence with an in‐person cohort induction, followed by regular supervised sessions, and supplemented by online check‐ins. They felt this would provide unscheduled opportunities for people with LTCs and their carers to meet individually, and in sharing learning and experiences, offer peer accountability and motivation. Professionals felt that with proper training and direction, digital tools could empower people with LTCs to become their own community navigators, capable of identifying and accessing PA resources from primary and community care.

My view is that in time what needs to happen is we all become community navigators. We all become social prescribers, and we all have that knowledge. The internet is a wonderful thing…and not being proprietorial about that knowledge, having that open source, putting it out there, freely available, brilliant, and making that as simple as possible. P4

(voluntary, community)

A significant barrier to upskilling the public in utilising digital tools to promote access to community‐based PA, was public service funding cuts. Local authority funding had been severely impacted at local and national levels, meaning that although well‐placed to support PA, community navigators faced workforce cuts. Due to funding cuts, community organisations were required to focus in on their qown resourcing, particularly in retaining experienced staff (including volunteers), maintaining facilities (including IT infrastructure) and providing their core services, as opposed to having the freedom to explore multisectoral partnerships and new interventions.

So our big challenges as are having enough finance to allow us to be resourced in terms of paid staff who require all the IT support, rent, payment for their salaries, training, etc, plus all the opportunity to recoup volunteers, retain volunteers and support volunteers and then actually being able to deliver the services to those individuals that we're looking after. P3

(voluntary, community)

### 7.2 Impact of Transport and Geographical Inaccessibility on Opportunities

For many, especially people with LTCs unable to drive, unemployed and/or receiving financial support, gym memberships were widely seen as ineffective in the long‐term. An addition for carers was the conflict in priorities, in dedicating funding to both travel and then organised PA, when money and time could be best spent elsewhere.

I think finances are a big thing when it comes to exercising…like if I go to a yoga class or something that I pay for, it's a really big treat…it's prioritising sometimes, like I think sometimes I have work, sometimes I need to go and see my mum, sometimes I want to see a friend…it is just trying to find that balance, isn't it? C5

(20 years, female)

People felt they needed to be more aware of PA and public health opportunities in their neighbourhoods, particularly near transport routes, to overcome travel costs and burdens. In terms of active travel, local geography impacted people's opportunities for PA via commuting, particularly those having to manage multimorbidities.

I live at the top of a very long dragging hill. I find I try and put off coming up and down… But I think how I live stops me from being more active. I use the car more because I think I can't get back up that hill. I mean I recently had my hip done so I'm not walking as well as I could, but I am walking really well. FG3

(LTC, 74 years, female, type ii diabetes/cardiovascular condition)

### 7.3 System and Political Burdens

Public funding cuts was a major issue for community organisations, particularly in losing skilled and knowledgeable workforce. For provision and support for PA, community and leisure professionals were required to charge for their services. This directly impacted people with LTCs and carers, as previously successful services and schemes were being cut without their consultation as service users; they felt this focused on the short‐term, overlooking the problem of sustaining people's activity long‐term.

They don't talk to the patient. I'm sure I can't be the only one with this issue. So, you know then looking at what could be done and resourced across the system, and you know funding is an issue that can hurt … you know where the savings would be found, if people could be really active, and working through that. FG1

(LTC, 45 years, female, neurological condition)

## 8 Discussion

### 8.1 The Current Context

This study provides greater understanding of the factors influencing progression along an intersectoral pathway between UK healthcare and community services, to support PA maintenance for people with LTCs and carers. We identified limited access to and information about appropriate PA services for people with LTCs; a need for improved intersectoral partnership; and key opportunities and barriers to developing a pathway for PA maintenance at a city‐level. People with LTCs had difficulties managing their conditions due to pain, fatigue and multimorbidities, which meant that their physical capabilities and motivation fluctuated over time. Limited signposting to community‐based PA opportunities was a major need, restricted access and ongoing support for people with LTCs. Time due to working, caring and finances, restricted access to organised PA for carers, therefore they sought routine in incidental, daily activities to maintain PA. Professionals faced local authority funding cuts, meaning that their own organisational goals and resourcing were prioritised over intersectoral collaboration to share expertise, resources and opportunities. This limited the support that they could offer to maintain PA, including supported self‐management and personalised care for LTC patients. Intermediaries, and digital tools linking to a centralised online hub, emerged as potential solutions to provide ongoing support, and improve signposting to appropriate, community‐based PA for people with LTCs. These offer scope for addressing system‐barriers, and in providing access to safe and/or supervised community‐based opportunities for PA and learning digital skills.

### 8.2 Challenges in Supporting Physical Activity (PA) Maintenance Across Healthcare and Community Settings

Structured PA pathways begin, primarily in healthcare with initial assessment, needs analyses and brief intervention(s), and subsequently ‘exit’ with signposting to community‐based PA [4, 46, 47]. The ‘*limited access to and information about PA services*’ theme highlights the need for improved signposting to community‐based PA and ongoing support, to support PA maintenance. Based on our screening survey, only 17% multisectoral professionals reported using behaviour change techniques to support people with LTCs in maintaining PA. The majority were intermediaries, such as health and wellbeing coaches, when following‐up support from healthcare consultations. Regarding solutions, there was little professional discussion about using behaviour change techniques and PA maintenance strategies (e.g. self‐monitoring to understand their individual PA behaviours, and goal setting to establish PA routines in daily life), as recommended by the literature to build autonomy and self‐efficacy [14, 48]. This concurs with a recent review which found little consensus in components used in PARS, but did highlight behaviour change as a potential key determinant for PARS success [16, 49]. Intermediary roles are still emerging, are largely UK and US‐centric, and contingent on national healthcare policies and clinical pathways [25, 29, 50]. For example, it is prerequisite for a UK Primary Care Network to have a social prescriber, but not a health coach. Although well‐placed and skilled, the effectiveness of intermediaries in initiating and maintaining PA for people LTCs remains unclear.

To maintain motivation, participants living with LTCs required signposting to age‐ and condition‐specific activities, including ongoing social support from professionals and peers. This concurs with cancer patients having undertaken PA programmes [51], and emphasises the importance of identifying activities that are appropriate to the individual's capabilities, in‐light of multimorbidities and symptoms, and exercise typologies (i.e., routinely active, intermittently active, or low active). Social support is integral to this, from professional guidance, peer companionship, accountability, to validation [52]. We found some LTC participants saw the value of being autonomous (i.e., being independently active without reliance on social group, or supervised activities at set times and locations). Autonomy support is undoubtedly an important component for future programmes [49]. For those who successfully maintain, PA becomes habit in their daily routine, with social support in different forms helping to sustain behaviour [48, 52].

### 8.3 Access to Community Physical Activity (PA) Opportunities Requires Effective Signposting and Professional Networks

A lack of appropriate opportunities, and limited awareness of local opportunities were two major barriers preventing people with LTCs and carers from maintaining behaviour either independently or socially. People with LTC expressed the need for PA appropriate to their conditions and age, which concurs with recent qualitative insights [14, 51]. However, health, social care and exercise professionals not well integrated into community networks, had limited awareness of local PA opportunities, which reduced their capabilities for signposting. McGrath et al. [31] highlighted the importance of primary care professionals being able to have person‐centred, therapeutic conversations to support their LTC patients being physically active. This includes active signposting to opportunities beyond primary care, and involving behaviour change techniques to develop self‐efficacy and promote social support, which were absent in this study. Professionals interviewed were challenged by public funding cuts, which not only reduced their resources, but also their skilled and knowledgeable workforce. Quality signposting required intersectoral professional networks. An example of which, health and wellbeing coaches were responsible for creating and managing networks alongside their roles. Professional networks also have value in terms of maintaining digital literacy amongst healthcare professionals, and promoting people with LTCs’ use of digital tools to maintain activity [41]. In this current study, people with LTCs and carers valued healthcare professionals and intermediaries being digitally trained to increase awareness in, and provide opportunities for community‐based PA.

The theme ‘*a need for improved intersectoral collaboration’* shows that communication and collaboration between services was inconsistent, inefficient and unintegrated, primarily due to financial constraints and organisational priorities (i.e., focusing on own resourcing). Collaborative working is necessary within ‘whole systems approaches’, at sectoral and organisational levels for PA promotion for people with LTCs [46]. This study's findings suggest that intersectoral collaboration is not happening across healthcare, social and voluntary sector organisations to support people with LTCs to maintain PA within their communities. Direct and active involvement of intermediaries may be crucial for establishing and sustaining future intersectoral collaborations, via their influence in engaging with both professionals and public at a community‐level.

### 8.4 Recommendations for Developing an Integrated Physical Activity (PA) Pathway

To strengthen progression along intersectoral pathways to support PA maintenance for people with LTCs, this research recommends that:

1. Primary healthcare and community services should prioritise efforts to signpost people with LTC to accessible, age‐ and condition‐appropriate PA opportunities. Where possible, these should incorporate behaviour change support as a routine component, to optimise personalised care. Involvement should include health and wellbeing coaches, and social prescribers, as professionals not only aware of community‐based options, but also capable of providing GPs and primary healthcare professionals with guidance for brief health conversations to support PA maintenance with people with LTCs.
2. Developing, resourcing and integrating intermediary roles, such as NHS Health and Wellbeing Coaches, is continued across sectors. Intermediaries can support people with LTCs, carers and professionals to identify and access community‐based opportunities, yet their effectiveness is untested for PA provision [30].
3. Intersectoral collaboration must be prioritised by service leaders, with time and ongoing resourcing dedicated to building and maintaining professional networks across healthcare, community and local authorities, to promote ongoing support. Collaborations should align to local public health priorities, and nationally align to national health service agendas (including digital strategies).
4. At a system level, investment in digital infrastructure could promote further intersectoral partnerships, increase visibility and signposting, and foster greater intersectoral professional and public communication.

### 8.5 Implications for Policy, Practice and Future Research

This study has identified key recommendations to inform the development of integrated pathways to facilitate PA from primary care for people with LTCs and carers. Future research should devise and test intersectoral interventions between healthcare and community services for PA maintenance. Interventions should focus on involving intermediaries to optimise signposting to appropriate opportunities, with which to build the capabilities and maintain motivation for people with LTCs and their carers. Co‐production with primary healthcare, voluntary community professionals, and LTC communities is vital, and can identify target behaviour change techniques and intervention strategies, based on the needs of people with LTCs and their carers.

Professionals should aim to deliver approaches balancing social support, with opportunity to be active independently. Previous work has shown these can help build confidence [51], develop self‐management skills, and promote PA being embedded into daily routines [14, 48]. The influence of social support and appropriate opportunities is established for PA maintenance. However, further evidence is needed to understand the differing needs of people with LTCs for low, intermittent and maintained PA typologies [51], and how interventions must adapt to address these needs to promote personalised care. Healthcare professionals should account for this when having health and wellbeing conversations with their patients living with LTCs.

This research also provides insights into professional needs in sharing expertise and resources between services, at a time of global economic and sociopolitical uncertainty, wherein health services are bringing care closer to people's communities. Integrated communication pathways contribute to this, but require ongoing management and maintenance. In the UK, the NHS’ Digital Transformation [53], aims to adopt technologies to optimise inter‐professional communication and efficiency in service access for patients. At present, this ‘digital’ agenda is in it's infancy, but in combination with the NHS’ Neighbourhood Health [25], offers potential for changing the way health services adopt PA in the prevention and management of LTCs, direct in people's local communities. Professionals should continue to create local inter‐professional networks, within their own organisational priorities (e.g., neighbourhood health), but with partners who offer novel expertise or insights (e.g., community‐based PA) to optimise care delivery.

### 8.6 Strengths and Limitations

This research benefitted from a series of public engagement, community cafes to disseminate our previous PARS‐focused work [14, 41] and recruit people with LTCs and their carers from under‐represented communities. These helped capture a range of public and multisectoral perspectives across an ethnically diverse, urban area. This included different health conditions (including people with multimorbidities), carers, and habitual PA, and professionals across sectors, localities and services. The community cafes were also successful method to recruit people with LTCs and their carers from underserved and deprived areas, particularly as 35% of our participants lived in the most deprived 10% of neighbourhoods in England [39]. The cafés success in recruitment may be attributable to the research team forging trusted partnerships with local community organisations and leaders, which offered understanding of the specific community's interests and needs. We also co‐produced the café evaluation methods with attendees, which help relationship‐building with potential participants. In data analysis, the COM‐B model [33] allowed us to acquire, and offer clear recommendations for services to develop and optimise integrated PA pathways, based upon the capabilities and opportunities of the service users and providers. However, the introduction of the Theoretical Domains Framework [54], would have afforded broader mapping of our findings beyond individual behaviour, to include professional practices, intersectoral partnership, and system‐level implementation challenges.

This study's limitations related to diversity in recruitment. Firstly, around two‐thirds of our participants with LTCs were white British, mostly female (76%) with over half retired. This likely limited insights from different cultures and socioeconomic groups, regarding access and engagement in PA services. Despite recruitment through underserved communities, and in partnership with multisectoral organisations, we could have involved community champions from various ethnic groups in the city to improve sample diversity. Secondly, while efforts were made to increase the perspectives from carers, we only managed to recruit five participants who were mostly educated, retired and white British. Our community cafes were advertised for people with LTCs, but not for carers, although they were eligible to attend. Additional recruitment drives were made during data analyses, yet we were unsuccessful in acquiring carer perspectives from less affluent areas and underserved communities. Future research should focus on the carers needs, particularly their relationship with their care recipient in maintaining PA in partnership. Finally, this study was based on a single, port city in southern England, and findings may differ from other communities, urban or rural, including those more centralised with fewer settled, migrant communities and less developed healthcare service infrastructure. This may have influenced themes relating accessibility to health services and community partnerships, in offering more varied perspectives based on health inequalities and cultural norms.

## 9 Conclusion

Recommendations arising from this study's findings suggest that PA pathways between healthcare and community services should focus on referral and signposting to appropriate, local physical activities involving social support from peers and professionals. To support PA maintenance, intermediaries play a crucial role in supporting patients’ and carers behaviour change, through their trusted and pivotal position interlinking primary care to community services. To optimise intersectoral partnership, professionals recommended a centralised digital hub for signposting local services, opportunities and skills sharing, and long‐term investment in community champions. Conversely, people with LTC and carers valued targeted advertisement via trusted, public settings, such as pharmacies and health centres.

## Author Contributions

**James P. Gavin:** conceptualisation, investigation, funding acquisition, writing – original draft, formal analysis, methodology, supervision, project administration, data curation. **Luisa Holt:** writing – review and editing, visualisation, software, formal analysis, data curation. **Xylo Chiu:** investigation, writing – review and editing, software, formal analysis, data curation, visualisation. **Charlotte Dando:** writing – review and editing, conceptualisation, investigation, funding acquisition, methodology. **Suzanne McDonough:** writing – review and editing, conceptualisation, resources.

## Ethics Statement

Ethical approval was granted by the University of Southampton's Faculty of Environmental and Life Sciences research ethics board (ERGO ref: 92231.A1). Consent was obtained from participants prior to interviews and focus groups, which were audio‐recorded and transcribed verbatim, with participants anonymised on transcription.

## Conflicts of Interest

The authors declare no conflicts of interest.

## Supporting information


Supporting File 1

Supporting File 2

Supporting File 3

Supporting File 4

Supporting File 5

Supporting File 6

Supporting File 7

Supporting File 8

## Data Availability

All data supporting the findings of this study are available in the supporting material of this article.
