# Supplementary material for: How Can We Help People With Long‐Term Health Conditions Understand and Engage in Physical Activity Maintenance From Healthcare to Community Settings? Perspectives of Patients, Carers and Professionals
Source: Health Expect. 2026 Nov 5;29(6):e70961. doi: 10.1111/hex.70961 (PMC13736783; doi:10.1111/hex.70961)
Supplement: Supplementary file 2 [file HEX-29-e70961-s001.docx]

Supplementary file 8. Themes, subthemes, summary of content, and illustrative quotes for people living with long-term conditions (LTCs), their carers, and multisectoral professionals: Opportunities and barriers to developing a pathway for physical activity (PA) maintenance.

| **Theme** | **Subtheme** | **Summary of content** | **Illustrative quotes** |
| --- | --- | --- | --- |
| 3. Opportunities and barriers to developing a pathway for PA maintenance | 3.1 Effect of limited funding and resources on opportunities | Public priorities | “*You've only got a set amount of money coming in, you don't ever have extra. So you're going to pull that from your food budget”* FG4 (LTC, 43 years, female, musculoskeletal condition)  *“Specific to Southampton, the financial condition of the* [organisation]... *So, we are seeing the teams that we need to work through shrink. That makes our lives difficult.”* P9 (local authority)  “*So our big challenges as are having enough finance to allow us to be resourced in terms of paid staff who require all the IT support, rent, payment for their salaries, training, etc, plus all the opportunity to recoup volunteers, retain volunteers and support volunteers and then actually being able to deliver the services to those individuals that we're looking after.”* P3 (voluntary, community) |
|  |  | Public seeking private healthcare | *“... these days the doctors are phone call consultations, but it's hard to see someone in person. I’ll give you an example, I have a very painful toe, and I think it's arthritis. So do I call the GP to look at my toe, or do I find someone else and fund it myself ... professional therapist, they’re quite pricey.”* FG3 (LTC, 74 years, female, type ii diabetes/cardiovascular condition) |
|  |  | Willingness to subsidise flexible PA | *“…£10 a week puts me off doing something. If it was £5 a week and I loved it, I would. I can't justify spending £40 or £50 on something for me in the month. If it was £20 then I would probably look after myself…you need things to support you*” C2 (58 years, female)  *“...we wish we could pay, cause it’s quiet, it’s very good. You buy a minimum, but this means they are there for support ... one time or twice a week. There needs to be instruction.”* FG4 (LTC, 48 years, female, COPD/cardiovascular condition) |
|  |  | Financial pressures limiting partnership working | *“Problems and barriers that are stopping these things* [integration of pathways] *happening often it does come down to funding.’* P1 (health and social care) |
|  | 3.2 Digital literacy | Online resources – cost-effective but burdensome | “*I do go online. It’s just you end up having your whole life online. I'm so slow and take it's an effort, I don't really enjoy it. I do it because I have to. You can learn everything now online ... but it's not going to get me active*. FG3 (LTC, 74 years, female, type ii diabetes) |
|  |  | Importance of professionals being digitally trained | *“If it was on an app, then obviously, social prescribers or GP would be like ‘have you checked this out?’ How do you check that out? And then obviously the onus is on you, but at least they've told you where to look”* FG4 (LTC, 48 years, female, COPD/cardiovascular condition) |
|  |  | Long-term solution – empowering multisectoral professionals and public as community navigators | *“My view is that in time what needs to happen is we all become community navigators. We all become social prescribers, and we all have that knowledge. The internet is a wonderful thing...and not being proprietorial about that knowledge, having that open source, putting it out there, freely available, brilliant, and making that as simple as possible.* P4 (voluntary, community) |
|  | 3.3 Impact of transport and geographical inaccessibility on opportunities | Increased awareness of local opportunities | “*...we need more sight of what goes on in our neighbourhoods. I'm sure there are groups out there in churches, community centres, maybe that we’ve not heard of. Accessible as well, because if they* [people] *have to use public transport, they're not going to get there*.” FG2 (LTC participant, 59 years, female, neurological condition)  *“...it's finding locations that are on public transport routes because not everyone can use taxis and not everybody who's got cars.”* FG2 (LTC, 59 years, female, neurological condition) |
|  |  | Local geography impacts active travel | *“I live at the top of a very long dragging hill. I find I try and put off coming up and down ... But I think how I live stops me from being more active. I use the car more because I think I can’t get back up that hill. I mean I recently had my hip done so I’m not walking as well as I could, but I am walking really well.”* FG3 (LTC, 74 years, female, type ii diabetes/cardiovascular condition) |
|  | 3.4 System and political burdens | Funding cuts and service charges | *“…community navigation…we have a system that works...essentially, it's vastly underfunded. So there's not many of us left doing it as it were, and that's been a process of cutbacks with the Council, particularly, not having any money to fund these services. So we don't have to reinvent the wheel as it were, it does happen already and we are doing it and it works but we just need more of it. Essentially, we need more resource to be able to do that”.* P4 (voluntary, community) |
|  |  | Services and schemes cut without public consultation | "*We had somebody called a physical activity navigator. This was a post that has since been cut. So when people contacted our navigators and said ‘I'm looking to do something’, if it was something involving in being active, looking to lose weight, or joining a sports club…they would be referred*" P4 (voluntary, community)  “*Yeah, a lot of places are pulling out of the money because the government or whoever organises it, are saying that they need to put the money elsewhere. They're pulling out because they used to be a lot of Sure Starts* [UK government scheme offering child and family health support] *when my kids were younger, a lot of them have shut down because there's no money*”. FG2 (LTC, 60 years, male, cardiovascular disease)  *“They don't talk to the patient. I'm sure I can't be the only one with this issue. So you know then looking at what could be done and resourced across the system, and you know funding is an issue that can hurt you know where the savings would be found if people could be really active and working through that.”* FG1 (LTC, 45 years, female, neurological condition) |
|  |  | Administrative/system burdens | “*I needed to get the sign off first from the GP partners... So that's where it can slow down a little bit, back and forth, just checking a few things over. Again, different surgeries do very different things, which is quite surprising because they're all in the same area*.” P13 (health and social care) |
