# Supplementary material for: How Can We Help People With Long‐Term Health Conditions Understand and Engage in Physical Activity Maintenance From Healthcare to Community Settings? Perspectives of Patients, Carers and Professionals
Source: Health Expect. 2026 Nov 5;29(6):e70961. doi: 10.1111/hex.70961 (PMC13736783; doi:10.1111/hex.70961)
Supplement: Supplementary file 3 [file HEX-29-e70961-s003.docx]

Supplementary file 6. Themes, subthemes, summary of content, and illustrative quotes for people living with long-term conditions (LTCs), carers and multisectoral professionals: Limited access to and information about appropriate physical activity (PA) services to support transition.

| **Theme** | **Subtheme** | **Summary of content** | **Illustrative quotes** |
| --- | --- | --- | --- |
| 1. Limited access to and information about appropriate PA services to support transition | 1.1 Experiences of people with LTCs and carers | Difficulties managing LTCs/fluctuating capability and motivation | *“...doing normal day-to-day tasks to find really tricky and challenging ...when I'm also dealing with increased pain levels trying to engage in some kind of arranged activity is just really overwhelming. I end up retreating and not doing anything ...”* FG1 (LTC, 62 years, female, type ii diabetes/osteoarthritis)  *“I have a chronic digestive disorder. That means I struggle with my energy levels. I have fatigue myself…then I also have osteoporosis and I know how important it is for that to walk”* C2 (58 years, female) |
|  |  | Significance of an increased risk of falling | *“I think it's just a thing with ageing ... I fall over a lot, but I'm too young for a falls clinic. They sent me for other investigations and never found anything* [and say] ... ‘*you can't do that because you're not… you shouldn't be in that position* [too young to fall]’*.”* FG4 (LTC, 43 years, female, musculoskeletal condition)  *“My balance is not that good. The traffic is too fast…you have to do what you can do to accommodate what things you can’t do.”* FG3 (LTC, 74 years, female, type ii diabetes/cardiovascular condition) |
|  |  | Motivation – peer and professional support is essential | “*If you've got a long-term health condition, it ain't going to get better. So, my motivation is going to go. I can see my motivation going downhill ... so you need the support, you need that*.” FG2 (LTC, 59 years, female, neurological condition)  “*Because if some* [exercise classes] *are socially related is that it gives the potential for a sensible and practical way to find a real peer or friend. I’d like to meet a peer on a non-pressure basis.”* C2 (58 years, female) |
|  |  | Importance of independence, awareness and reflection | *“Some people say yeah. ‘You’re going on your own!?’ People the same age as me. Some people say I couldn’t do that, or my husband won’t let me.*” FG3 (LTC, 81 years, female, musculoskeletal condition)  “*I think my main motivation is exercise is like the way I come into my body and my brain. I get a lot of feedback, that I'm not able to get in my day-to-day life, when I’m running around.* [Reflecting on] …*how I’m actually doing physically and emotionally.”* C5 (20 years, female) |
|  |  | LTCs and caring limiting access to opportunities | *“...the majority of my friends don't have conditions ... I can't keep up with them. So it's quite lonely ... I had somebody with a similar condition, it might be a little bit easier, but then again there the issue with that is that, if they're really struggling on the day, or I'm struggling on the day, then we're not going to go. It's not having that motivation from somebody else that you're exercising along with.”* FG1 (LTC, 62 years, female, type ii diabetes/ osteoarthritis)  “*Let's say that I have another member of my family* [helping me] *and I don't have so much to do…then I take the time that I’d rather spend doing activities, to then either catch up on work, sleep, whatever I need to do instead of sports*” C4 (78 years, male) |
|  | 1.2 Need for ongoing support | Limited information and signposting to PA services | *“And there was almost nothing from the NHS in the way of advice or support like that. They simply said ‘Come back to us if you have a problem. We'll get in touch in six months to see how you're doing’. Which they didn't.*” C3 (50 years, male)  *“Information? Nobody knows where to go, there’s no point that says, 'these are all the things that are available to you’.”* FG3 (LTC, 81 years, female, musculoskeletal condition)  *“... you just get cut off* [after rehab]*. That's demoralising. Where do I go now?* *Did they go, 'this is where you can go for a walk with these people who don't wanna carry on with exercises.’ If they're going to get you to do a 6 week or 3-month thing. ... they work with you for the first few months, then they say ‘we can recommend you go somewhere else’, in the last month.”* FG2 (LTC, 60 years, male, cardiovascular condition) |
|  | 1.3 Approaches to improve community-based opportunities for PA | Appropriate physical activities | “*...they had a sort of movement and dance which was much simpler, less intensive level where most of the people much older than me and it was for people with osteoporosis*.” FG1 (LTC, 52 years, female, musculoskeletal condition)  *“...when I was referred through the exercise referral scheme, I got sent to a gym and the instructor just gave me really hard exercises without really understanding anything…it made the pain worse. You know, I didn't want to carry on going because I could barely move*.” FG1 (LTC, 45 years, female, neurological condition) |
|  |  | Options and signposting to opportunities | *“...you obviously want options. I think there's fear involved. I mean … you need to be reminded of what you can do. It's about confidence as well. I fall a bit like my friend over there, and it really knocks your confidence. It's maybe knowing, for certain conditions, what the top three exercises are. If you've got diabetes, top three exercises*.” FG2 (LTC, 51 years, female, respiratory condition/obesity)  “*I forgot to mention another community-based thing, they organise walks for different levels of abilities. They have yoga classes, Pilates classes, they have various group-based activities, social as well as physical activity. I think that kind of thing is possibly helpful and if you can't get somewhere. I don't like driving... there will be someone who they sort of pair up, so there will be someone who'll drive you wherever you need to go.”* FG1 (LTC, 50 years, female, type ii diabetes/musculoskeletal condition)  *“I think both having a self-referral and a professional referral works really well to be able to give that patient that option...So having both of those routes are really important I feel to increase that start rate.”* P1 (health and wellbeing coach) |
|  |  | Promotion via primary care and local public services | “*I was gonna say the GP surgery would be the best place to find that information. Because everyone knows what a medical practice is, don't they*?” FG3 (LTC, 74 years, female, type ii diabetes/cardiovascular condition)  “*The one thing we lack is actively advertising that we have this GP referral service and the patients we can help. I'm working with our marketing co-ordinator to try and get some marketing to be put back into both surgeries, so they make our patients aware of what's going on*.” P6 (health and personal trainer)  “*We’re not encouraged to put up posters* [in public places] *and things like that which I think makes it difficult to reach different groups of the community...so yeah, it's really hard, but we don't have good links like that in the city.”* P5 (voluntary, community) |
|  |  | Community settings to promote peer interaction (social opportunities) | “*Church is a nice, mutual, comfortable place to go. But it's people, you know…it's helping that person to go out, and then they might come next time around because they'll be curious. So doing meetings, physical meetings*.” FG2 (LTC, 57 years, female, type ii diabetes)  *“…you'd want somewhere local enough. I often find if it's going to be a 20-minute drive, I think, ‘Is that realistic? Am I going to keep doing that?’. If there were things more local that would, you know* [be active], *there is more opportunity to stick to it*.” C2 (58 years, female) |
|  |  | Building psychological capability of healthcare professionals | “*From a clinician who’s sat on the chair, they’re face-to-face with the patient, I think their confidence and their skills to have conversations is really important. I had a referral from a GP recently…a real prime example of a GP saying ‘I don't know where to start and what to advise them’...I did find some resources, which were designed for GP's in consultation to, if your patient has RA* [rheumatoid arthritis] *or COPD, then this is how you would have that conversation. It was lovely because it even broke it down into if you had 2-minutes, 1-minute, 5-minutes*.” P2 (health and wellbeing coach)  *“...if we can provide them with as much information as possible, then we're able to answer any question that the patient comes to us [with]... we're able to help increase their confidence. I think it's about us as professionals and having that network to be able to go out and talk to these providers, to go to those sessions, to get all of the answers to these questions, as much as it is for the providers to create this information*.” P1 (health and wellbeing coach) |
|  |  | Building physical capability of participants – recognising the importance of planning and goal setting | *“... if it's something that's regular, you can plan it in because like if I've got something on, I know that like depending on what it is, because I don't always know what the kickback is going to be, like fatigue wise.”* FG4 (LTC, 48 years, female, COPD/cardiovascular condition)  *“I've got adrenal failure... here is that element of chronic fatigue and up and down all day long, every day ... the steroids last and make me feel normal for about an hour and a half each time I take them and that's three times a day. So I try and fit in what I can during that time and then the rest of the day.”* FG1 (LTC, 63 years, female, musculoskeletal condition) |
|  |  | Improved ongoing support - opportunities in their local environment and continuing social connections | *“You go to rehab and you get to a state of fitness and then it stops. You do 12 sessions over six weeks plus 12 sessions for as long as it takes you and. At the end of that, it's like. Yeah. And I don't have. I don't have the motivation to do it on here.”* FG2 (LTC, 60 years, male, cardiovascular condition)  “*You know, with depression and that the group, you know, to help you get in six weeks. Six weeks and then you're just left. Oh, you can come back, but it's got to be 3 months or six months or something.*” FG2 (LTC, 52 years, female, musculoskeletal condition)  *“You can start the course off with like an in person, you know, like group thing. So, there is that face to face ... seeing* [people] *on the screens after those, and then maybe once a month meet up. You've still got that social thing, and you can obviously make the decision yourselves to meet up.”* FG4 (LTC, 48 years, female, COPD/cardiovascular condition) |
|  | 1.4 Approaches to improve online opportunities for PA | Guidance using digital tools – people with LTCs and professionals | “*I think knowing your audience, because even when things change, e-Consult to Admiral or whatever it's called now. Some people don't use technology that often. I mean* [I am] *mixed generation, but even I find it frustrating using that stupid system, not being able to ring the doctor to do something for you, rather than just say, ‘do you want me to talk you through it?’ Which must be frustrating for them*.” FG4 (LTC, 68 years, male, type ii diabetes/COPD)  “*I think the problem is that you know there is a digital gap in the city and that a lot of older people don't have access to the things I've just mentioned... and those are the people that you know we need to be linking with health and well-being coaches, social prescribers, community navigators, voluntary sector organisations to help and encourage them to start moving more*.” P16 (voluntary, community)  “…particularly exercise prescription software that allows the patient to communicate back, can be really helpful”. P17 (health and social care) |
