# Supplementary material for: How Can We Help People With Long‐Term Health Conditions Understand and Engage in Physical Activity Maintenance From Healthcare to Community Settings? Perspectives of Patients, Carers and Professionals
Source: Health Expect. 2026 Nov 5;29(6):e70961. doi: 10.1111/hex.70961 (PMC13736783; doi:10.1111/hex.70961)
Supplement: Supplementary file 4 [file HEX-29-e70961-s004.docx]

**Supplementary file 4** Focus group guide (people with LTCs) / Interview guide (carers).

1. **Introductions**

- Welcome participants and thank them for attending
- Explain focus group / interview ground rules to the attendees
- Remind participants that the focus group will be audio-recorded and video-recorded (if online)
- Ask if they have any questions or concerns before proceeding and remind them to keep the discussions confidential.
- Introductions – please introduce yourself and briefly tell us why you are interested in this research.

Purpose of the focus group – We are interested in hearing about your experiences of being physically active alongside managing your long-term condition(s) between primary healthcare (e.g., your GPs) and community services. We would also like to gain your views about our plans to support the development of a pathway in the city of Southampton to improve partnership working between different organisations that support people to remain physically active.

Purpose of the interview – We are interested in hearing about your experiences of being physically active as a carer of someone living with a long-term condition(s) between primary healthcare (e.g., your GPs) and community services. In particular, we want to know about your experiences of staying physically active and helping your partner, family or friend living with a LTC stay active. We would also like to gain your views about our plans to support the development of a pathway in the city of Southampton to improve partnership working between different organisations that support people to remain physically active.

2. **Current physical activity behaviour**

- Currently, are you as physically active as you would like to be?

Prompt - what stops you doing this?

Carers only – how do you support your partner/family member/friend to stay physically active whilst living with an LTC?

- If you are active, how do you manage to stay active from day to day?

If not, what needs to happen to make regular physical activity easier? Carers only - Particularly as a carer?

Prompt – do you feel confident in your abilities to keep active?

- What support or resources do you need to help you remain physically active?

Prompt – are you physically active with others? Do other people give you support? practical/encouragement etc from family/friends/Health Care Professinals (HCPs)/exercise professionals

- What local community-based opportunities are there to help you move more?

3. **Views on a physical activity pathway**

A physical activity pathway can be thought of as a well-structured roadmap to help guide people toward a more active lifestyle. It involves a coordinated approach to providing services, information, guidance, and opportunities for physical activity (PA) in a local area. We have already identified different parts of the pathway and would like to hear your thoughts about what things would make it easier/harder for you to engage in each stage.

- Entry points (referral/access)
- Triage/tailoring/personalised support (assessment/identify activities) with menu of opportunities to choose activities.
- PA opportunities and delivery
- Exit routes/signposting/ongoing support – confident to be independently active/need more/less/different support.

What support would be helpful throughout these stages? (Behaviour change support) (e.g. goal setting, coping plans, reviews, appropriately trained staff).

Any stages or parts of the pathway that you feel we have missed?

4. **Anything to add?**

- Is there anything you would like to add to what we have talked about but not yet discussed?
