# Supplementary material for: How Can We Help People With Long‐Term Health Conditions Understand and Engage in Physical Activity Maintenance From Healthcare to Community Settings? Perspectives of Patients, Carers and Professionals
Source: Health Expect. 2026 Nov 5;29(6):e70961. doi: 10.1111/hex.70961 (PMC13736783; doi:10.1111/hex.70961)
Supplement: Supplementary file 5 [file HEX-29-e70961-s005.docx]

**Supplementary file 2.** Descriptor of components necessary for a physical activity pathway, used to supplement the interview and focus group guides.

Triage

Behavioural Support

Opportunities

Developed by Move Consulting^35^, based on the findings of the preceding NIHR Maintenance Of physical acTivity beHaviours (MOTH) programme, specific to exercise referral schemes^14^ and digital tools^40^ to promote physical activity (PA) maintenance for people with long-term conditions (LTCs).
