# Supplementary material for: How Can We Help People With Long‐Term Health Conditions Understand and Engage in Physical Activity Maintenance From Healthcare to Community Settings? Perspectives of Patients, Carers and Professionals
Source: Health Expect. 2026 Nov 5;29(6):e70961. doi: 10.1111/hex.70961 (PMC13736783; doi:10.1111/hex.70961)
Supplement: Supplementary file 6 [file HEX-29-e70961-s006.docx]

**Supplementary file 1.** Example community café agenda.

**ACTIVE LIVING COMMUNITY CAFÉS**

**Day** 23^rd^ April 9.30 –11.30 am

**Location** Thornhill Baptist Church

**AGENDA**

| **TIME** | **ACTIVITY** |
| --- | --- |
| 9.30-9.45 | Registration & refreshments  Welcome & introductions  Ground rules |
| 9.45-10.15 | Educational (research) talk   - Lessons learnt from MOTH* Findings - Q&A |
| 10.15-11.15 | Open forum   - What local community-based opportunities are there to help you move more? - How can we help you move more in your community? - How would you like to see NHS/GP surgeries working with community providers to help you stay active? - What made you come and join this programme? What would make you join? - What made you stay involved with this programme? What would make you stay? |
| 11.15-11.30 | Evaluation/pledges, sharing study recruitment materials,  chat & coffee, |

 *MOTH, Maintenance Of physical acTivity beHaviours.

Feedback will be sought from attendees and organisers regarding community cafés as a knowledge exchange and public engagement method.

**How the public benefit?**

i. Learning on how to better self-manage using physical activity.
ii. Opportunity to share their experiences and potential to evaluate local services for physical activity.
iii. Meet new people and socialise.
iv. Opportunity to reflect on their journey
v. Opportunity to be involved in contemporary research, relating to public health
