# Supplementary material for: How Can We Help People With Long‐Term Health Conditions Understand and Engage in Physical Activity Maintenance From Healthcare to Community Settings? Perspectives of Patients, Carers and Professionals
Source: Health Expect. 2026 Nov 5;29(6):e70961. doi: 10.1111/hex.70961 (PMC13736783; doi:10.1111/hex.70961)
Supplement: Supplementary file 7 [file HEX-29-e70961-s007.docx]

**Supplementary file 3.** Interview guide (professionals).

1. **Introduction & check consent**

Interview consent – discuss interviewee’s consent to be interviewed:

- Check interviewee is happy to go ahead with the interview
- Check they have been through the informed consent process
- Check interviewee is happy for interview to be audio and video-recorded
- Ask if they have any questions or concerns before proceeding and remind them to keep the discussions confidential

Purpose of the interview – We are interested in hearing about your experiences of working in a service that supports people to remain physically active alongside managing their LTCs (from an intersectoral perspective involving primary healthcare and community organisations). We would also like to gain your views about our plans to support the development of a pathway in the city of Southampton to improve partnership working between different organisations that support people to remain (or ‘maintain’) physically active.

2. **Supporting physical activity (PA) behaviours**

- How do you support people with LTCs to remain physically active?

Prompt – how do you help people to develop the skills and confidence they need to move more often in a way that works for them?

- What things help you to carry out this role? What things make it harder?

Prompt – have you had any formal training regarding PA and behaviour change

- What local community-based opportunities do you know of for people with LTCs to help them move more?

3. **Views on a physical activity pathway**

A physical activity pathway can be thought of as a well-structured roadmap to help guide people toward a more active lifestyle. It involves a coordinated approach to providing services, information, guidance, and opportunities for physical activity in a local area. We have already identified different parts of the pathway (see the five PA pathway components) and would like to hear your thoughts about what things would make it easier/harder for you to engage in each stage.

- Entry points (referral/access)
- Triage/tailoring/personalised support (assessment/identify activities) with menu of opportunities to choose activities
- PA opportunities and delivery
- Exit routes/signposting/ongoing support – confident to be independently active/need more/less/different support

What support would be helpful throughout these stages? (behaviour change support) (e.g., goal setting, coping plans, reviews, appropriately trained staff).

Any stages or parts of the pathway that you feel we have missed?

- If there is not already a pathway in place, what do you think is stopping the creation of a PA pathway? (or what would help it happen)

4. **Anything to add?**

Is there anything you would like to add to what we have talked about but not yet discussed?
