# Supplementary material for: How Can We Help People With Long‐Term Health Conditions Understand and Engage in Physical Activity Maintenance From Healthcare to Community Settings? Perspectives of Patients, Carers and Professionals
Source: Health Expect. 2026 Nov 5;29(6):e70961. doi: 10.1111/hex.70961 (PMC13736783; doi:10.1111/hex.70961)
Supplement: Supplementary file 8 [file HEX-29-e70961-s008.docx]

Supplementary file 7. Themes, subthemes, summary of content, and illustrative quotes for people living with long-term conditions (LTCs), their carers, and multisectoral professionals: A need for improved intersectoral collaboration within a pathway.

| **Theme** | **Subtheme** | **Summary of content** | **Illustrative quotes** |
| --- | --- | --- | --- |
| 2. A need for improved intersectoral collaboration within a pathway | 2.1 Improved intersectoral communications | Siloed working | “*It's really hard, but we don't have good links like that in the city... I don't ever really hear anything about the [event] and that seems like a missed opportunity to work together and to spread that message further*.” P5 (voluntary, community)  *“The wider secondary care team? I think it's more difficult to access and not always a prompt response...*” P19 (health and social care) |
|  |  | Local and city networks | *“We've set up for ourselves a health and wellbeing coach local network ...So, if a new group comes up for, say physical activity, then we are able to share that across the network.”* P2 (health and social care) |
|  | 2.2 Importance of established and trusted links with intermediaries | Lack of public awareness but valuable | “*Where do you find these social prescribers*.” FG3 (LTC, 80 years, female, type ii diabetes)  *“She was really lovely…putting strategies in place for, you know, tasks, and life and general and mental health and whatever. But then kind of once they were in place, it was kind of like ‘we were doing really well. So you can always come back, you know…there's obviously going to be a limit.”* FG4 (LTC, 43 years, female, musculoskeletal condition) |
|  |  | Limited professional awareness of intermediary roles | *“…I'm fairly digitally oriented, being able to find things and communicate…the GP should be a route, and if you like, social prescribers…a prescriber network may help.”* C4 (78 years, male)  *“I think having more knowledge of what is around the city and what things are going on. One of my managers invited me to go to a green social prescribing event a few weeks ago in the town centre, and I met* [name] *who works for* [organisation]*, and he was talking about the things they're doing. I wasn't aware that they’ve got a physical activity social prescribing course you can do and networking with him meant that I’m going to do that course…it’s about who you know, rather than what you know.* P6 (health and personal trainer) |
|  | 2.3 Community partnerships - understanding the experiences of services and LTC communities | Integrating PA into public health | “*There's a community group ... they have a what they call a coffee and cake, right? But it's mostly, it's church based,* [its] *very popular. So if you have somebody who's talk about activities, what they could do that might be in place*.” FG3 (LTC, 74 years, female, type ii diabetes/cardiovascular condition) |
|  |  | LTC peer development schemes | *“…using friends, actually people that are like minded who want to do things with you…as well having that extra person to encourage you and always say ‘right I'm gonna meet you there’*. FG2 (LTC, 82 years, male, musculoskeletal condition)  *“Because if something* [pathway/scheme] *is socially related, that it gives potential for a sensible and practical way to find a real peer or friend. I mean slightly different, but … chat to people going in … you check one another”* C2 (58 years, female) |
|  | 2.4 Understanding experiences of other services and LTC communities | Public sharing experiences and opportunities | *“…somewhere where we've all got something unique to ourselves. ‘I've got this place to go to today. Maybe I should help that person’. Great, because there isn't anything out there that's easy to access where you feel safe.”* FG2 (LTC, 60 years, male, cardiovascular condition)  *“People have long-term conditions, but people have so much to offer. If you volunteer, for example … the great thing about volunteering is that you are immediately helping others and you're being positive and doing something really useful. That switch of mindset is outstanding, you can make a massive difference to people's lives. They suddenly get back a sense of who they are and what they're about and lose that stigma of having a long-term condition.”* P4 (voluntary, community) |
|  |  | Local knowledge and intersectoral working to optimise personalised care | *“…we're able to get a real clear idea of potentially what we can support them with... it's about knowing local area, knowing what opportunities there*.” P1 (health and social care)  *‘We work purposefully with a roundabout up to a hundred different organisations. So we may be looking after shared people and they need different areas of specialism.”* P3 (voluntary, community) |
